# Medication patterns in older adults with multimorbidity: a cluster analysis of primary care patients

**DOI:** 10.1186/s12875-019-0969-9

**Published:** 2019-06-13

**Authors:** Marina Guisado-Clavero, Concepción Violán, Tomàs López-Jimenez, Albert Roso-Llorach, Mariona Pons-Vigués, Miguel Angel Muñoz, Quintí Foguet-Boreu

**Affiliations:** 1 Gerència d’Àmbit d’Atenció Primària Barcelona Ciutat, Institut Català de la Salut, Carrer Balmes 22, Barcelona, Spain; 2 Institut Universitari d’Investigació en Atenció Primària Jordi Gol (IDIAP Jordi Gol), Gran Via Corts Catalanes, 587 àtic, 08007 Barcelona, Spain; 3 Universitat Autònoma de Barcelona, Plaça Cívica, 08193 Bellaterra, Barcelona, Spain; 4 Facultat d’infermeria, Universitat de Girona, Emili Grahit, 77, 17071 Girona, Spain; 5 Unitat de Suport a la Recerca de Barcelona, Institut Universitari d’Investigació en Atenció Primària Jordi Gol (IDIAP Jordi Gol), Carrer Sardenya, 375, 08025 Barcelona, Spain; 6 Departament de psiquiatria, Hospital Universitari de Vic, Francesc Pla el Vigatà, 1, 08500 Vic, Barcelona, Spain; 7 Facultat de Ciències de la Salut i Benestar, Universitat de Vic – Universitat Central de Catalunya, Sagrada Família, 7, 08500 Vic, Spain

**Keywords:** Ageing, Cluster analysis, Drugs, Electronic health records, Multimorbidity, Primary health care

## Abstract

**Background:**

Older adults suffer from various chronic conditions which make them particularly vulnerable. The proper management of multiple drug use is therefore crucial. The aim of our study was to describe drug prescription and medication patterns in this population.

**Methods:**

A cross-sectional study in Barcelona (Spain) using electronic health records from 50 primary healthcare centres. Participants were aged 65 to 94 years, presenting multimorbidity (≥2 chronic diseases), and had been prescribed at least 1 drug for 6 months or longer during 2009. We calculated the prevalence of prescribed drugs and identified medication patterns using multiple correspondence analysis and k-means clustering. Analyses were stratified by sex and age (65–79, 80–94 years).

**Results:**

We studied 164,513 patients (66.8% women) prescribed a median of 4 drugs (interquartile range [IQR] = 3–7) in the 65–79 age-group and 6 drugs (IQR = 4–8) in the 80–94 age-group. A minimum of 45.9% of patients aged 65–79 years, and 61.8% of those aged 80–94 years, were prescribed 5 or more drugs. We identified 6 medication patterns, a non-specific one and 5 encompassing 8 anatomical groups (alimentary tract and metabolism, blood, cardiovascular, dermatological, musculo-skeletal, neurological, respiratory, and sensory organ).

**Conclusions:**

Drug prescription is widespread among the elderly. Six medication patterns were identified, 5 of which were related to one or more anatomical group, with associations among drugs from different systems. Overall, guidelines do not accurately reflect the situation of the elderly multimorbid, new strategies for managing multiple drug uses are needed to optimize prescribing in these patients.

**Electronic supplementary material:**

The online version of this article (10.1186/s12875-019-0969-9) contains supplementary material, which is available to authorized users.

## Introduction

Worldwide, individuals are living longer [1] thanks to advances in medical research and care [2]. For instance, in 2016, 19% of the European population was aged 65 years or older [3], a figure that is expected to reach 30% by 2060 [4]. Nevertheless, a longer life span is closely related to the likelihood of developing chronic disease [5] and 55–98% of older adults suffer from multimorbidity [6]. Such patients are more likely to require multiple drugs to achieve optimal clinical (or disease) management [7, 8], indeed, a prescription rate of over 80% for ≥5 drugs has been reported [9]. Multiple drug use in older adults, however, is associated with overall worsening physical and psychological health as a result of age-related changes in pharmacokinetics and pharmacodynamics [10]. In addition, it has a potential influence on aspects of safety, including inappropriate prescription, adverse drug reaction, risk of medication interaction (drug-drug or drug-disease interaction), and adherence [11, 12].

Due to ageing vulnerability, multiple drug use in the multimorbid elderly is a main issue of concern for the public health system. Identifying which drugs are being taken is crucial to define patients at risk. As a result, tools need to be developed with the aim of decreasing prescription errors, drugs interactions, adverse drug reactions, and other consequences such as falls, hospitalization, and mortality associated with multiple drug use [13, 14]. A recent systematic review described clinical management oriented to multimorbidity and polymedication. Its recommendations, however, were focused on the risks/benefits of each drug individually rather than collectively [15]. To date, the limited information available in the literature is mostly descriptive [16] and methods regarding pharmaco-epidemiology in multimorbidity have yet to be established. Prescription groups and patterns could be of help in the analysis of multiple drug use to create new strategies in the management of complexity among multimorbid patients.

New techniques are being developed to create homogeneous patterns regarding the management of prescribed drugs. For instance, exploratory factor analysis (EFA) which is based on correlations between variables or factors, and cluster analysis (CA), a technique for grouping a set of individuals in such a way that they are more similar to each other than those in other groups [17]. EFA has recently been reported to be useful for describing correlation between variables, while CA carries out an in-depth examination of the pattern for non-random associations between the determinant variables of an individual [18]. In recent years, EFA has been employed to define a number of multimorbidity patterns [19–21], and some medication ones [22]. Nonetheless, the statistical technique employed should be taken into account. EFA correlates specific variables (e.g. diseases), but not all the variables of one unit (e.g. patient), whilst CA could be helpful as the main starting point to look for dissimilarities. Irrespective of the methodology employed in these studies [23], there are common biological systems encompassing multimorbidity patterns: cardio-metabolic conditions, musculoskeletal diseases, and mental health problems [24]. Serious diseases and those with a greater prevalence according to EFA/CA should thus be represented with the corresponding medication.

We hypothesized that prescribed drugs could be grouped using CA to identify clusters of patients with similar drugs and consequently create medication patterns. The objective of this study was to describe prescribed drugs and identify medication patterns in multimorbid older adults.

## Methods

### Design, setting, and inclusion criteria

**Fig. 1:**
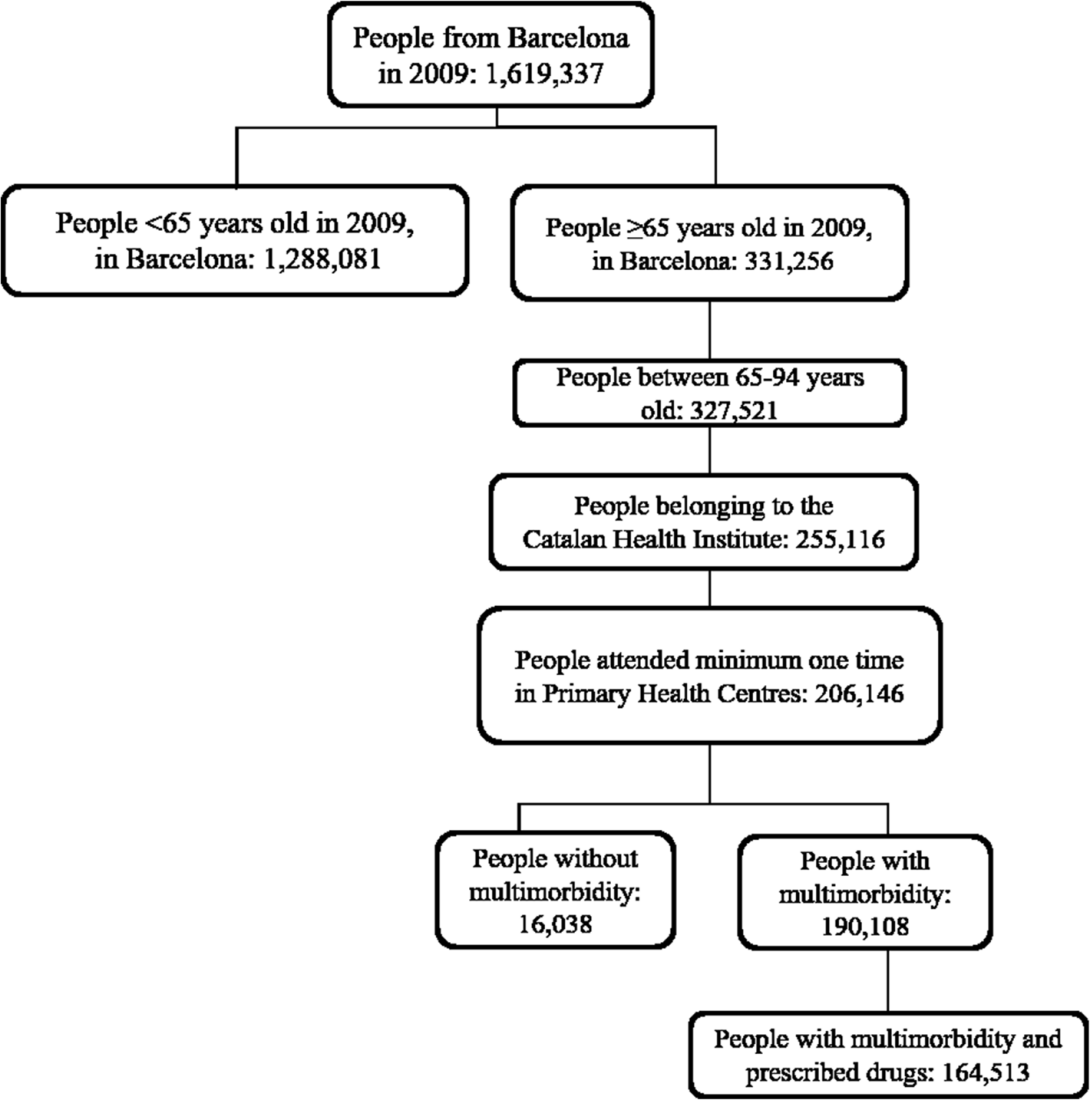
Flow chart

We conducted a cross-sectional analysis of electronic health records (EHR) from the Information System for Research in Primary Care (SIDIAP). This is a centralized database that contains EHR from 2006 for all the patients who have attended primary health care centres (PHCC) run by the public Catalan Health Institute [25, 26]. The study was performed in Barcelona (Spain) in 2009 with information from 50 PHCC. The participants were aged 65 to 94 years, and the inclusion criteria were a) to have attended a PHCC at least once during 2009; b) to present multimorbidity, defined as the coexistence of 2 or more chronic diseases [27]; and c) to have been prescribed at least 1 drug for a period of 6 months or longer during 2009 (see flow chart in Fig. 1).

The study protocol was approved by the Research Ethics Committee at IDIAPJGol (Protocol no: P15/149). All data were anonymized, and the confidentiality of the EHR was maintained at all times in accordance with national and international law. As all data were anonymized, no consent to individuals were required.

### Variables

Prescription drugs were the main unit of measurement and were coded as 1 (present) or 0 (absent). Drugs in the SIDIAP database are classified using the Anatomical Therapeutic Chemical (ATC) system (Additional file 1), a measuring unit recommended by the World Health Organization for drug studies. To classify the drugs in this study, and facilitate subsequent analysis and interpretation, we used the 4th level of the ATC system which corresponds to chemical subgroups. Proton pump inhibitors, for example, are coded as A02BC [28].

The other variables recorded for each participant were: number of chronic diseases coded with the International Classification of Primary Care second edition and selected using the O’Halloran criteria [29], age (65–79 years vs 80–94 years), and sex (male vs female). According to the chronic diseases selected, chronic medication was defined as the prescription of a drug for at least 6 continuous months during the period of study. Medication which did not fulfil this criterion was not analysed as it was considered acute or not long-term. Neither were supplements included as they are not financed by the Spanish health system.

### Statistical analysis

Data were extracted from the SIDIAP database after authorization of the study [25]. All the authors had access to the database. There were no missing values, as sex, age, chronic diseases, and drugs were recorded for all the sample.

Descriptive statistics were employed to summarize the overall data. Categorical variables were expressed as frequencies (percentage) and continuous variables as means (standard deviation [SD]) or medians (interquartile range [IQR]). Prevalence of prescription drugs was calculated and medication patterns identified through 2 steps: 1) multiple correspondence analysis (MCA), and 2) k-means clustering. All analyses were stratified by sex and age.

#### Multiple correspondence analysis

MCA is a data analysis technique used to detect and represent underlying structures in sets of nominal categorical data. It identifies groups with similar characteristics and shows, in a multidimensional space, relationships between dichotomous or categorical variables (in our case drug prescriptions) that would be difficult to observe in a contingency table [30, 31]. MCA also allows individuals to be directly represented as points (coordinates) in a geometric space through the transformation of original binary data to continuous ones. The MCA was based on the indicator matrix. The optimal number of dimensions extracted and percentages of inertia were determined by means of a scree plot.

#### K-means clustering

Using the geometric space created in the MCA, patients were classified into clusters according to proximity criteria by means of the k-means algorithm, and centers obtained for each cluster. The optimal number of clusters (k), which is the solution with the highest Calinski-Harabaz index value, was assessed using criteria with 100 iterations. To assess internal cluster quality, cluster stability of the optimal solution was computed using Jaccard bootstrap values with 100 runs [17]. Highly stable clusters should yield average Jaccard similarities of 0.85 and above.

### Medication patterns

To describe the medication patterns across the clusters, we used three criteria: a) the prevalence of prescribed drugs in each cluster; b) the observed/expected (O/E) ratios obtained by dividing the prevalence of a particular drug in each cluster by the prevalence of the same prescribed drug in the age and sex groups, considering over-represented drugs when value ≥2; and c) exclusivity, defined as the proportion of individuals with a particular prescribed drug included in the cluster over the total number of individuals with a particular prescribed drug in the corresponding age and sex group, considering high exclusivity when value ≥50%.

Medication patterns were defined by considering drugs with a prevalence ≥20% or an O/E ratio ≥ 2. To identify the importance of each medication and, as a consequence, the amount of medication included in a cluster, we employed exclusivity. In order to facilitate the designation of a medication pattern we named the patterns considering medications belonging to the same ATC group with an exclusivity value ≥50%, even when presenting a low prevalence. And we also took into consideration to name the pattern those drugs over-represented by O/E ratio ≥ 2. We then described medications included in each cluster using three numbers of characteristics: prevalent drugs (prevalence ≥20%), drugs over-represented (O/E ratio ≥ 2) and exclusive drugs (exclusivity ≥50%). But we considered only exclusive and over-represented drugs to label the pattern.

In addition to mathematical validation, clinical criteria based on previous literature [32–34] and clinical feedback from the research team (3 family physicians and 2 epidemiologists) were employed to evaluate the consistency and significance of the final cluster solution.

The analyses were carried out using SPSS for Windows, version 24 (SPSS Inc., Chicago, IL, USA) and R version 3.4.2 (R Foundation for Statistical Computing, Vienna, Austria).

## Results

The sample was composed of 164,513 patients aged ≥65 years all of whom presented multimorbidity and had at least 1 drug prescribed; 66.8% were women. The group 65–79 years had a mean age of 72.0 years (SD = 4.3) and was prescribed a median of 4 (IQR = 3–7) drugs. The group 80–94 years had a mean age of 84.1 years (SD = 3.4) and was prescribed a median of 6 (IQR: 4–8) drugs. At least 45.9% of the 65–79 year and 61.8% of the 80–94 year groups were prescribed 5 or more drugs. As expected, the use of 10 or more drugs was almost twice in the 80–94 compared to the 65–79 year age group. The number of prescribed drugs and chronic diseases did not differ between sexes (Table 1). The 10 most widely prescribed drugs across the sample belonged to 3 ATC system groups: alimentary tract and metabolism (A), nervous system (N), and cardiovascular system (C). *Proton pump inhibitors* and *HMG CoA reductase inhibitors* were present in the top 3 most prescribed drugs in all groups, with *platelet aggregation inhibitors (excluding heparin)* in men and *benzodiazepine derivatives* (65–79 years) and *anilides* (80–94 years) for women (Table 2).

**Table 1:** Descriptive data, by sex and age groups, of the multimorbid patients (*n* = 164,513) aged 65–94 years attended in 2009 at primary healthcare centres located in Barcelona

	Women		Men	
	65–79 years	80–94 years	65–79 years	80–94 years
Participants n (%)	78,008 (47.4)	31,848 (19.4)	41,931 (25.5)	12,726 (7.7)
Number drugs n (%)				
1–4	40,931 (52.5)	11,374 (35.7)	22,703 (54.1)	4868 (38.3)
5–9	31,500 (40.4)	16,460 (51.7)	16,339 (39.0)	6268 (49.3)
≥10	5577 (7.1)	4014 (12.6)	2889 (6.9)	1590 (12.5)
Median number of drugs (IQR^a^)	4 (3–7)	6 (4–8)	4 (2–6)	5 (3–8)
Number of chronic diseases n (%)				
2	2806 (3.6)	1125 (3.5)	1792 (4.3)	410 (3.2)
[3–5]	20,301 (26.0)	7689 (24.1)	12,484 (29.8)	3090 (24.3)
[6–9]	33,089 (42.4)	13,495 (42.4)	17,955 (42.8)	5562 (43.7)
≥10	21,812 (28.0)	9539 (30.0)	9700 (23.1)	3664 (28.8)
Median number of chronic diseases (IQR^a^)	7 (5–10)	8 (5–10)	7 (5–9)	7 (5–10)

*IQR*^a^ Interquartile range

**Table 2:** The ten most commonly prescribed drugs in 2009 for multimorbid patients (*n* = 164,513) aged 65–94 years, by sex and age groups, attended at primary healthcare centres located in Barcelona

	Women				Men			
	ATC code^a^	Drug name	N	%	ATC code^a^	Drug name	N	%
65–79 years	A02BC	Proton pump inhibitors	32,634	41.8	C10AA	HMG CoA reductase inhibitors	18,188	43.4
	C10AA	HMG CoA reductase inhibitors	32,004	41.0	A02BC	Proton pump inhibitors	15,170	36.2
	N05BA	Benzodiazepine derivatives	20,649	26.5	B01AC	Platelet aggregation inhibitors excl. Heparin	13,872	33.1
	N02BE	Anilides	18,434	23.6	C09AA	ACE inhibitors, plain	9748	23.2
	B01AC	Platelet aggregation inhibitors excl. Heparin	15,338	19.7	G04CA	Alpha-adrenoreceptor antagonists	7235	17.3
	C09AA	ACE inhibitors, plain	13,578	17.4	A10BA	Biguanides	6306	15.0
	M05BA	Bisphosphonates	13,309	17.1	N05BA	Benzodiazepine derivatives	6019	14.4
	N06AB	Selective serotonin reuptake inhibitors	11,522	14.8	C08CA	Dihydropyridine derivatives	5996	14.3
	C03AA	Thiazides, plain	10,112	13.0	C07AB	Beta blocking agents, selective	5934	14.2
	C09CA	Angiotensin II antagonists, plain	9231	11.8	N02BE	Anilides	5625	13.4
80–94 years	A02BC	Proton pump inhibitors	16,496	51.8	A02BC	Proton pump inhibitors	5877	46.2
	N02BE	Anilides	11,370	35.7	B01AC	Platelet aggregation inhibitors excl. Heparin	5641	44.3
	C10AA	HMG CoA reductase inhibitors	11,222	35.2	C10AA	HMG CoA reductase inhibitors	4657	36.6
	B01AC	Platelet aggregation inhibitors excl. Heparin	10,512	33.0	C09AA	ACE inhibitors, plain	3235	25.4
	N05BA	Benzodiazepine derivatives	9633	30.2	N02BE	Anilides	2638	20.7
	C09AA	ACE inhibitors, plain	7223	22.7	G04CA	Alpha-adrenoreceptor antagonists	2601	20.4
	C08CA	Dihydropyridine derivatives	5283	16.6	N05BA	Benzodiazepine derivatives	2313	18.2
	C03CA	Sulfonamides, plain	5265	16.5	C08CA	Dihydropyridine derivatives	2260	17.8
	N06AB	Selective serotonin reuptake inhibitors	5258	16.5	C03CA	Sulfonamides, plain	1930	15.2
	C09CA	Angiotensin II antagonists, plain	4502	14.1	C01DA	Organic nitrates	1622	12.7

Code^a^: chemical subgroup, 4rt level, ATC code (Anatomical Therapeutic Chemical classification) from the World Health Organization (Additional file 1)

For more details, visit webside: https://www.whocc.no/atc/structure_and_principles/

### Characteristics of medication patterns

Six medication patterns for each age and sex group were identified. All the groups had a *non-specific* pattern consisting of highly prevalent drugs that were neither over-represented nor exclusive. The other 5 patterns were made up of drugs belonging to 1 or more anatomical groups corresponding to: *alimentary tract and metabolism* (A), *blood and blood forming organs* (B), *cardiovascular system* (C), *dermatological* (D), *musculoskeletal system* (M), *nervous system* (N), *respiratory system* (R), and *sensory organs* (S) (Table 3, Additional files 2, 3 and 4).

**Table 3:** Example of medication patterns across women 65–79 years attended in primary health centres in Barcelona during 2009 (*N* = 78,008)

	Code^&^	Drugs	Pre^b^	O/E ratio^a^	Exclus.
Cluster 1 *n* = 39,202 (50%)					
Non-specific pattern	C10AA	HMG CoA reductase inhibitors	32%	0.78	39%
	A02BC	Proton pump inhibitors	21%	0.51	26%
Cluster 2 *n* = 14,604 (19%)					
Nervous system pattern	A02BC	Proton pump inhibitors	70%	1.68	31%
	N05BA	Benzodiazepine derivatives	58%	2.18	41%
	C10AA	HMG CoA reductase inhibitors	56%	1.36	25%
	N06AB	Selective serotonin reuptake inhibitors	40%	2.71	51%
	B01AC	Platelet aggregation inhibitors excl. Heparin	35%	1.76	33%
	M05BA	Bisphosphonates	28%	1.62	30%
	N02BE	Anilides	26%	1.10	21%
	N06AX	Other antidepressants	14%	3.49	65%
	N03AX	Other antiepileptics	10%	3.16	59%
	N05CD	Benzodiazepine derivatives	9%	2.03	38%
	A12AA	Calcium	7%	2.58	48%
	N06AA	Non-selective monoamine reuptake inhibitors	7%	3.07	57%
	C01DA	Organic nitrates	7%	2.32	43%
	N02AX	Other opioids	6%	2.03	38%
	A11CC	Vitamin D and analogues	6%	2.97	56%
	A06AD	Osmotically acting laxatives	6%	2.04	38%
	N03AE	Benzodiazepine derivatives	4%	3.69	69%
	C07AA	Beta blocking agents, non-selective	4%	2.61	49%
	H02AB	Glucocorticoids	4%	2.71	51%
	C10AX	Other lipid modifying agents	4%	2.25	42%
	N06DX	Other anti-dementia drugs	3%	2.44	46%
	A03FA	Propulsive	3%	2.25	42%
Cluster 3 *n* = 9502 (12%)					
“Musculo-skeletal system” and “Dermatologicals” pattern	A02BC	Proton pump inhibitors	66%	1.58	19%
	N02BE	Anilides	61%	2.57	31%
	N05BA	Benzodiazepine derivatives	33%	1.26	15%
	M02AA	Antiinflammatory preparations, non-steroids for tropical use	31%	5.96	73%
	C10AA	HMG CoA reductase inhibitors	30%	0.74	9%
	M01AE	Propionic acid derivatives	27%	4.30	52%
	C05CA	Bioflavonoids	19%	2.88	35%
	M01AX	Other antiinflammatory and antirheumatic agents, non-steroids	17%	2.84	35%
	A02AD	Combinations and complexes of aluminium, calcium and magnesium compounds	15%	4.24	52%
	M01AB	Acetic acid derivatives and related substances	15%	5.17	63%
	D01AC	Imidazole and triazole derivatives	10%	5.93	72%
	N02AX	Other opioids	10%	3.25	40%
	D07AC	Corticosteroidas, potent (group III)	9%	6.65	81%
	N02BB	Pyrazolones	6%	5.30	65%
	A06AD	Osmotically acting laxatives	6%	2.13	26%
	R05CB	Mucolytics	5%	2.83	34%
	R06AX	Other antihistamines for systemic use	5%	3.34	41%
	N07CA	Antivertigo preparations	5%	2.26	28%
Cluster 4 *n* = 8745 (11%)					
Alimentary tract and metabolism pattern	C10AA	HMG CoA reductase inhibitors	68%	1.67	19%
	A10BA	Biguanides	65%	5.86	66%
	B01AC	Platelet aggregation inhibitors excl. Heparin	61%	3.12	35%
	A02BC	Proton pump inhibitors	50%	1.19	13%
	A10BB	Sulfonylureas	37%	6.69	75%
	C09AA	ACE inhibitors, plain	30%	1.70	19%
	C08CA	Dihydropyridine derivatives	29%	2.68	30%
	C07AB	Beta blocking agents, selective	23%	2.18	24%
	N02BE	Anilides	22%	0.94	10%
	A10AC	Insulins and analogues for injection, intermediate-acting	10%	6.34	71%
	C01DA	Organic nitrates	9%	3.22	36%
	A10AE	Insulins and analogues for injection, long-acting	9%	6.04	68%
	M04AA	Preparations inhibiting uric acid production	9%	2.97	33%
	C02CA	Alpha-adrenoreceptor antagonists	7%	3.57	40%
	C10AB	Fibrates	7%	2.96	33%
	A10AD	Insulins and analogues for injection, intermediate- or long- acting combined with fast- acting	7%	6.24	70%
	G04CA	Alpha-adrenoreceptor antagonists	4%	2.13	24%
	C10AX	Other lipid modifying agents	4%	2.36	26%
Cluster 5 *n* = 3275 (4%)					
Respiratory system pattern	R03AC	Selective beta-2-adrenoreceptor agonists	72%	16.88	71%
	A02BC	Proton pump inhibitors	54%	1.30	5%
	R03BB	Anticholinergics	54%	18.86	79%
	R03AK	Adrenergics in combination with corticosteroids or other drugs, excl. Anticholinergics	51%	11.87	50%
	R03BA	Glucocorticoids	40%	18.45	77%
	C10AA	HMG CoA reductase inhibitors	38%	0.94	4%
	N02BE	Anilides	32%	1.36	6%
	N05BA	Benzodiazepine derivatives	27%	1.01	4%
	B01AC	Platelet aggregation inhibitors excl. Heparin	23%	1.17	5%
	C03CA	Sulfonamides, plain	14%	2.11	9%
	R05CB	Mucolytics	11%	6.34	27%
	R06AX	Other antihistamines for systemic use	6%	3.79	16%
	C08DB	Benzothiazepine derivatives	5%	2.26	9%
	H02AB	Glucocorticoids	4%	3.07	13%
	G04CA	Alpha-adrenoreceptor antagonists	4%	2.02	8%
Cluster 6 *n* = 2680 (3%)					
Cardiovascular system pattern	B01AA	Vitamin K antagonists	78%	15.74	54%
	C03CA	Sulfonamides, plain	66%	10.05	35%
	A02BC	Proton pump inhibitors	58%	1.37	5%
	C01AA	Digitalis glycosides	53%	27.60	95%
	C10AA	HMG CoA reductase inhibitors	43%	1.06	4%
	N02BE	Anilides	32%	1.35	5%
	N05BA	Benzodiazepine derivatives	29%	1.11	4%
	C09AA	ACE inhibitors, plain	27%	1.56	5%
	C07AB	Beta blocking agents, selective	24%	2.25	8%
	C09CA	Angiotensin II antagonists, plain	23%	1.98	7%
	A12BA	Potassium	20%	16.35	56%
	C03DA	Aldosterone antagonists	19%	17.02	58%
	C08DB	Benzothiazepine derivatives	13%	6.07	21%
	C07AG	Alpha and beta blocking agents	12%	8.91	31%
	M04AA	Preparations inhibiting uric acid production	11%	3.73	13%
	C01DA	Organic nitrates	9%	3.26	11%
	B03AA	Iron bivalent, oral preparations	5%	3.60	12%
	A10AE	Insulins and analogues for injection, long-acting	3%	2.30	8%
	A02BA	H2 - receptor antagonists	3%	2.12	7%

Code^&:^Chemical subgroup, 4rt level, ATC code (Anatomical Therapeutic Chemical classification) from the World Health Organization

O/E ratio^a^: Observed/expected ratio

Pre^b^: Prevalence

Exclus.: Exclusivity

Selected criteria: Prevalence ≥ 20 or Observed/Expected ratio ≥ 2

As an example, findings for women 65–79 years are represented in Table 3. Six medication patterns were identified, numbered according to the weight of the sample implied (descending order): *non-specific* (cluster 1), followed by *nervous system* (cluster 2), *musculo-skeletal + dermatological* (cluster 3), *alimentary tract and metabolism* (cluster 4), *respiratory system* (cluster 5), and *cardiovascular system* (cluster 6). For each cluster, three subgroups of prescribed drugs that encompassed the pattern were defined. Three kinds of data were shown for every cluster. Using the example of the *musculo-skeletal and dermatological* pattern (cluster 3), we identified three different groups of drugs in the pattern:

a) drugs with a high prevalence but not over- represented such as *proton pump inhibitors* (prevalence 66%, O/E ratio 1.58, exclusivity 19%) and *benzodiazepine derivatives* (prevalence 33%, O/E ratio 1.26, exclusivity 15%);

b) drugs with a high/low prevalence over-represented with exclusivity < 50% such as *anilides* (prevalence 61%, O/E ratio 2.57, exclusivity 31%) and *other opioids* (prevalence 10%, O/E ratio 3.25, exclusivity 40%);

c) drugs with a high/low prevalence over-represented and with exclusivity ≥ 50% such as *anti-inflammatory preparations, non-steroids for topical use* (prevalence 33%, O/E ratio 5.96, exclusivity 70%) and *potent corticosteroids (group III)* (prevalence 9%, O/E ratio 6.65, exclusivity 81%) (Table 3).

It was observed that the *non-specific* pattern had the greatest number of patients for all groups and was defined by drugs that were neither prevalent nor over-represented. With respect to the *non-specific* pattern, the number of patients aged 65–79 years was higher than those aged 80–94 years for both sexes. According to the frequency of patients, the next patterns were: for women 65–79 years “*nervous system*” and “*musculo-skeletal + dermatological*”, whilst for women 80–94 years they included *alimentary tract and metabolism* as a drug group implied in frequency; for men 65–79 years they were “*cardiovascular system*” and “*alimentary tract and metabolism*”, and for those 80–94 years was added the drug group related to *musculo-skeletal* and *nervous system* (Table 3, Additional files 2, 3 and 4).

Comparing patterns between sexes, women had four patterns in both age groups which implied only one over-represented anatomical system (*alimentary tract and metabolism*, *cardiovascular system*, *nervous system*, and *respiratory system*), in contrast to men who had only two patterns implying one anatomical system (*cardiovascular* and *respiratory system*). The other patterns were formed by two or more anatomical systems. The rest of the results are detailed in Table 3 and Additional files 2, 3 and 4.

Comparing patterns between age groups, no significant differences were observed for women with the exception of additional drugs encompassing the *non-specific* pattern (*anilides*, *ACE inhibitors*, *benzodiazepine derivatives*) (Table 3, Additional file 2). The men’s patterns, however, appeared more complex: to the *non-specific pattern* were added two drugs (*platelet aggregation inhibitors excluding heparin* and *proton pump inhibitors*), and in the 80–94 age group the patterns encompassed multiple anatomical groups including a *sensory organs* pattern (Additional files 3 and 4).

## Discussion

In this study, we present data regarding prescription drugs in an urban population of elderly adults with multimorbidity. Prescription rates were high, particularly in the older subset of patients, probably due to the greater burden of chronic disease. Proton pump inhibitors were the most widely prescribed drug with cardiovascular and neurological drugs representing the most frequently prescribed groups. We defined 6 medication patterns which provide information about the multiple drugs grouped closely together in elderly patients. The pattern with the most participants, *non-specific*, had up to 39% of the age-sex sample included and was composed of drugs corresponding to specific diseases (hypertension, lipid disorder, depressive disorder (women)) and others related to the secondary prevention of cardiovascular/digestive diseases (platelet aggregation inhibitors and proton pump inhibitors). The rest of the medication patterns could be linked to the multimorbidity ones defined in a previous article performed in the same sample [35].

### Comparison with published literature

Ageing is associated with functional decline, and the prescription of multiple drugs tends to be highest in the oldest segments of the population [36]. Just over half the patients in our study had been prescribed 5 or more drugs, rates of between 45.0 and 80.0% have been previously described based on primary care EHR [9, 37]. These results showed that the 10 most prescribed drugs were to treat metabolic, cardiovascular, and nervous system disorders, in line with other reports for the elderly [35, 38, 39]. As expected, considering that heart disease is the leading cause of death in such populations [40], cardiovascular drugs were the main group of prescribed drugs. Looking more closely, proton pump inhibitors were the most widely prescribed drug in our study, contrasting with findings on the prevalence of digestive tract chronic diseases conducted in the same sample [35]. Off-label use of proton pump inhibitors could be related to the prevention of adverse gastrointestinal effects, as reported elsewhere [41]. In addition, a high prevalence of lipid modifying (C10AA) agents and antithrombotic drugs (B01AC) was probably linked to their use in the primary and secondary prevention of thrombotic events. We would like to point out that benzodiazepines, despite their potentially adverse effects for older adults (e.g, memory impairment, delirium, falls) [42, 43], were still frequently prescribed in our population (from 14.4% in men 65–79 years to 30.2% in women 80–94 years), with a reported prevalence among the elderly from 10.0 to 41.6% [44, 45].

Six patterns per group defining user profiles with prescribed drugs were obtained. We took into account prescribed drugs, instead of consumed ones, because we assumed patients followed what their doctors suggested. As we studied patients with multimorbidity, we considered chronic drugs rather than supplements or acute prescriptions. As a result, many of the defined patterns seemed logical and in concordance with chronic disease prevalence [35]. In addition, differences in intra- and inter-patterns were represented defining prevalence, O/E ratio, and exclusivity for each drug. The relevance of the prescribed drug was thus represented by these three parameters.

The *non-specific* pattern had the greatest number of patients in all strata as no anatomical group was over-represented. It could, therefore, be hypothesized that patients evolve to 5 specific patterns across time, that is to say, the *non-specific* pattern could represent a pre-state of a specific one. In addition, the fact that the number of patients included in the *non-specific* pattern was lower in the 80–94 than the 65–79 year group points to the hypothesis that this pattern could be a *pre-specific medication* one. Nevertheless, longitudinal analyses should be conducted to substantiate this issue. With respect to specific patterns, the men’s appeared more complex than women’s possibly because of the anatomical systems involved and male smoking habits [46]. In concordance with this difference, more men in the 65–79 year group presented *cardiovascular* and *respiratory* patterns than women who showed mostly *neuromuscular drug-related* patterns. Furthermore, the fact that the patterns of the older participants were made up of more than one anatomical system was possibly related to the burden of chronic disease associated with age [23]. The observed medication patterns should coincide with the multimorbidity ones given that the former reflect the various illnesses being treated. For instance, if we compare multimorbidity and medication patterns from the same sample, the *endocrine-metabolic multimorbidity* pattern should be related to the *alimentary tract and metabolism* one [35]. A concept that concurs with a number of publications that have reported that medication data may represent a way of identifying chronic conditions [47]. Following this idea, medication patterns could help characterise individuals with multimorbidity. Finally, the use of three criteria to define patterns permitted a representation of all drugs, including those related to low prevalence diseases. Variability between chronic diseases and treatments was thus respected in our results.

To the best of our knowledge, only one study has previously defined medication patterns using EFA [22], and few authors have investigated such patterns in patients with multimorbidity [16]*.* It is difficult to draw comparisons because of differences in drug inclusion criteria, number of drugs considered, and especially methodology. Nevertheless, some anatomical systems, including cardiovascular, respiratory, and neurological ones were the same. Such similarities are probably related to the strong prevalence of chronic conditions. Nevertheless, with CA we obtained 6 markedly different patterns, and with the O/E ratio and the exclusivity criteria we could define which drugs were over-represented, playing a more crucial role.

A recent publication has established that guidelines addressing polymedication appear arbitrary [15]. Our research thus contributes to the definition of medication patterns which could be used to identify both user profiles and safety issues (e.g. detecting prescription errors, for instance inappropriate drugs, or drug-drug associations), something that is not possible with multimorbidity patterns. The definition of medication patterns could open new paths to create instruments to prioritize groups of individuals and permit effective prescription. In addition, establishing medication patterns in accordance to multimorbidity patterns would help to determine prognostic factors in drug safety, define possible adverse drug reactions, and identify drug-drug and drug-disease interactions. The analysis of medication patterns thus provides an additional perspective for interpreting and defining the population’s health.

### Strength and weakness

Our study sample is both reliable and representative of the population, thus adding robustness to our results. Moreover, we provide an accurate reflection of real prescribing habits for the elderly with multimorbidity in an urban public primary health care setting. Analyses of individual medication patterns can lead to new insights into individual prescription situations. We consider that complexity among patients is well represented in these patterns. However, some limitations should be considered. On one hand, selected criteria of chronicity (prescription of 6 or more months) may have caused a selection bias, although we followed an established definition [23]. In addition, we have to assume that CA is inherently exploratory in nature and different clustering algorithms may produce varying results. The lack of studies defining medication patterns also limits comparisons between results and populations. Finally, we should consider as a limitation the fact that the collected data were 10 years old and may not exactly reflect current prescription patterns. Nevertheless, these medication patterns correspond to a six-year longitudinal multimorbidity study [35, 48] in which it was observed that multimorbidity patterns did not differ at all during the period studied. In addition, in public primary health care, the implementation of new treatments for specific diseases (for example, oral anticoagulants or oral antidiabetic medications) are not yet generalised. For this reason, we considered that the medication patterns represented current prescription.

### Future research

Medication patterns could change with time as a consequence of multimorbidity evolution and new treatments applied in some chronic diseases. Our study is cross-sectional, but in future research it would be advantageous to analyse large prospective cohorts with different estimates to define medication patterns and identify their stability or evolution. In addition, generational differences are expected due to modified lifestyle habits. Thus, re-analyses should be considered as medication patterns are expected to alter across decades.

Taking into account drug prescription and medication patterns, improvements in guidelines for the clinical management of elderly patients should be contemplated. In addition, the methodology used for clustering could be a starting point for analysing drug safety in relation to drug interaction.

## Conclusions

This study provides information about prescription drugs in an urban population of older adults with multimorbidity. Our results showed highly elevated prescription rates, particularly in the older subset of patients, probably due to the greater burden of chronic disease. Clinical practice should consider reviewing off-label prescribed drugs for possible de-prescription.

The study of medication patterns provides a method for analysing the use of multiple drugs in elderly patients. We identified 6 medication patterns in our series which could provide new avenues for evaluating multimorbidity.

## Additional files

Additional file 1: The main groups of the Anatomical Therapeutic Chemical (ATC) system. (DOCX 12 kb)

Additional file 2: Medication patterns across women 80-94 years attended in primary health centres in Barcelona during 2009 (*N* = 31,848). Selected criteria: Prevalence ≥20 or Observed/Expected ratio ≥2. (DOCX 24 kb)

Additional file 3: Medication patterns across men 65-79 years attended in primary health centres in Barcelona during 2009 (*N* = 41,931). Selected criteria: Prevalence ≥20 or Observed/Expected ratio ≥ 2. (DOCX 26 kb)

Additional file 4: Medication patterns across men 80-94 years attended in primary health centres in Barcelona during 2009 (*N* = 12,726). Selected criteria: Prevalence ≥20 or Observed/Expected ratio ≥ 2. (DOCX 23 kb)

## Data Availability

The data that support the findings of this study may be obtained from SIDIAP but restrictions could apply to those used under license. Upon reasonable request and with permission of SIDIAP they may be available from the authors.

## Abbreviations

ATC: Anatomical Therapeutic Chemical
CA: Cluster analysis
EFA: Exploratory factor analysis
EHR: Electronic health records
Exclus: Exclusivity
IDIAPJGol: Institut Universitari d’Investigació en Atenció Primària Jordi Gol
IQR: Interquartile range
MCA: Multiple correspondence analysis
O/E ratios: Observed/expected ratios
PHCC: Primary health care centres
Pre: Prevalence
SD: Standard deviation
SIDIAP: System for Research in Primary Care

## References

[1] Murray CJL, Barber RM, Foreman KJ, Ozgoren AA, Abd-Allah F, Abera SF, et al. Global, regional, and national disability-adjusted life years (DALYs) for 306 diseases and injuries and healthy life expectancy (HALE) for 188 countries, 1990–2013: quantifying the epidemiological transition. Lancet. 2015:1990–2013. 10.1016/S0140-6736(15)61340-X PMC4673910 26321261

[2] Oeppen J, Vaupel JW (2002). Demography. Broken Limits to Life Expectancy. Science (80- ).

[3] Eurostat. Population structure and ageing - statistics explained. 2016. http://ec.europa.eu/eurostat/statistics-explained/index.php/Population_structure_and_ageing. Accessed 23 Aug 2017.

[4] Eurostat. Active ageing - employment, social affairs & inclusion - European Commission. 2015. http://ec.europa.eu/social/main.jsp?catId=1062. Accessed 23 Aug 2017.

[5] Jacob L, Breuer J, Kostev K (2016). Prevalence of chronic diseases among older patients in German general practices. GMS Ger Med Sci.

[6] Marengoni A, Angleman S, Melis R, Mangialasche F, Karp A, Garmen A (2011). Aging with multimorbidity: a systematic review of the literature. Ageing Res Rev.

[7] Van den AM, Buntinx F (1998). Multimorbidity in general practice: prevalence, incidence, and determinants of co-occurring chronic and recurrent diseases. J Clin Epidemiol.

[8] Wehling M (2009). Multimorbidity and polypharmacy: how to reduce the harmful drug load and yet add needed drugs in the elderly? Proposal of a new drug classification: fit for the aged: letters to the editor. J Am Geriatr Soc.

[9] Freund J, Meiman J, Kraus C (2013). Using electronic medical record data to characterize the level of medication use by age-groups in a network of primary care clinics. J Prim Care Community Heal.

[10] Mangoni AA, Jackson SHD (2003). Age-related changes in pharmacokinetics and pharmacodynamics: basic principles and practical applications. Br J Clin Pharmacol.

[11] Hajjar ER, Cafiero AC, Hanlon JT (2007). Polypharmacy in elderly patients. Am J Geriatr Pharmacother.

[12] Salazar JA, Poon I, Nair M (2007). Clinical consequences of polypharmacy in elderly: expect the unexpected, think the unthinkable. Expert Opin Drug Saf.

[13] Gómez C, Vega-Quiroga S, Bermejo-Pareja F, Medrano MJ, Louis ED, Benito-León J (2015). Polypharmacy in the elderly: a marker of increased risk of mortality in a population-based prospective study (NEDICES). Gerontology..

[14] Oscanoa TJ, Lizaraso F, Carvajal A (2017). Hospital admissions due to adverse drug reactions in the elderly. A meta-analysis. Eur J Clin Pharmacol.

[15] Muth C, Blom JW, Smith SM, Johnell K, Gonzalez-Gonzalez AI, Nguyen TS, et al. Evidence supporting the best clinical management of patients with multimorbidity and polypharmacy: a systematic guideline review and expert consensus. J Intern Med. 2019;285(3):272–88. 10.1111/joim.12842 30357955

[16] Doos L, Roberts EO, Corp N, Kadam UT (2014). Multi-drug therapy in chronic condition multimorbidity: a systematic review. Fam Pract.

[17] Everitt BS, Landau S, Leese M, Stahl D. Cluster analysis. 5th ed: In John Wiley & Sons, Ltd; 2011. p. 321–30.

[18] Roso-Llorach A, Violán C, Foguet-Boreu Q, Rodriguez-Blanco T, Pons-Vigués M, Pujol-Ribera E (2018). Comparative analysis of methods for identifying multimorbidity patterns: a study of ‘real-world’ data. BMJ Open.

[19] Britt HC, Harrison CM, Miller GC, Knox SA (2008). Prevalence and patterns of multimorbidity in Australia. Med J Aust.

[20] Steinman MA, Lee SJ, John Boscardin W, Miao Y, Fung KZ, Moore KL (2012). Patterns of multimorbidity in Eldery veterans. J Am Geriatr Soc.

[21] Foguet-Boreu Q, Violán C, Rodriguez-Blanco T, Roso-Llorach A, Pons-Vigués M, Pujol-Ribera E (2015). Multimorbidity patterns in elderly primary health care patients in a South Mediterranean European region: a cluster analysis. PLoS One.

[22] Calderón-Larrañaga A, Gimeno-Feliu LA, González-Rubio F, Poblador-Plou B, Lairla-san José M, Abad-Díez JM (2013). Polypharmacy patterns: unravelling systematic associations between prescribed medications. PLoS One.

[23] Violan C, Foguet-Boreu Q, Flores-Mateo G, Salisbury C, Blom J, Freitag M (2014). Prevalence, determinants and patterns of multimorbidity in primary care: a systematic review of observational studies. PLoS One.

[24] Prados-Torres A, Calderón-Larrañaga A, Hancco-Saavedra J, Poblador-Plou B, Van Den Akker M (2014). Multimorbidity patterns: a systematic review. J Clin Epidemiol.

[25] García-Gil MDM, Hermosilla E, Prieto-Alhambra D, Fina F, Rosell M, Ramos R (2011). Construction and validation of a scoring system for the selection of high-quality data in a Spanish population primary care database (SIDIAP). Inform Prim Care.

[26] Sistema d'informació per al desenvolupament de la Investigació en Atenció Primària.Inicio. http://www.sidiap.org/index.php/es. Accessed 6 Feb 2018.

[27] Valderas JM, Starfield B, Sibbald B, Salisbury C, Roland M (2009). Defining comorbidity: implications for understanding health and health services. Ann Fam Med.

[28] Christian Berg Ms, Hege Salvesen Blix Ms, Irene Litleskare Ms, Marit Rønning Ms, Solveig Sakshaug Ms, Hanne Strøm Ms, et al. Guidelines for ATC classification and DDD assignment 2013. 16th editi. Oslo; 2013. 1-284 p. https://www.whocc.no/filearchive/publications/1_2013guidelines.pdf. Accessed 25 Jul 2017.

[29] O’Halloran J, Miller GC, Britt H (2004). Defining chronic conditions for primary care with ICPC-2. Fam Pract.

[30] Sourial N, Wolfson C, Zhu B, Quail J, Fletcher J, Karunananthan S (2010). Correspondence analysis is a useful tool to uncover the relationships among categorical variables. J Clin Epidemiol.

[31] García-Gil M, Blanch J, Comas-Cufí M, Daunis-i-Estadella J, Bolíbar B, Martí R (2016). Patterns of statin use and cholesterol goal attainment in a high-risk cardiovascular population: a retrospective study of primary care electronic medical records. J Clin Lipidol.

[32] Evidence-based clinical decision support at the point of care | UpToDate. https://www.uptodate.com/home. Accessed 23 Jul 2017.

[33] Cochrane Library. https://www.cochranelibrary.com/search. Accessed 21 Jul 2017.

[34] Guies de pràctica clínica. Institut Català de la Salut. http://ics.gencat.cat/ca/assistencia/coneixement-assistencial/guies-de-practica-clinica/. Accessed 10 Sept 2017.

[35] Guisado-Clavero M, Roso-Llorach A, López-Jimenez T, Pons-Vigués M, Foguet-Boreu Q, Muñoz MA (2018). Multimorbidity patterns in the elderly: a prospective cohort study with cluster analysis. BMC Geriatr.

[36] Payne RA, Avery AJ, Duerden M, Saunders CL, Simpson CR, Abel GA (2014). Prevalence of polypharmacy in a Scottish primary care population. Eur J Clin Pharmacol.

[37] Buck MD, Atreja A, Brunker CP, Jain A, Suh TT, Palmer RM (2009). Potentially inappropriate medication prescribing in outpatient practices: prevalence and patient characteristics based on electronic health records. Am J Geriatr Pharmacother.

[38] Slabaugh SL, Maio V, Templin M, Abouzaid S (2010). Prevalence and risk of polypharmacy among the elderly in an outpatient setting. Drugs Aging.

[39] Mizokami F, Koide Y, Noro T, Furuta K (2012). Polypharmacy with common diseases in hospitalized elderly patients. Am J Geriatr Pharmacother.

[40] Jackson CF, Wenger NK (2011). Cardiovascular disease in the elderly. Rev Española Cardiol (English Ed).

[41] George CJ, Korc B, Ross JS (2008). Appropriate proton pump inhibitor use among older adults: a retrospective chart review. Am J Geriatr Pharmacother.

[42] Gurwitz JH, Field TS, Harrold LR, Rothschild J, Debellis K, Seger AC (2003). Incidence and preventability of adverse drug events among older persons in the ambulatory setting. JAMA..

[43] Maher RL, Hanlon J, Hajjar ER (2014). Clinical consequences of polypharmacy in elderly. Expert Opin Drug Saf.

[44] Madhusoodanan S, Bogunovic OJ (2004). Safety of benzodiazepines in the geriatric population. Expert Opin Drug Saf.

[45] Morgan SG, Weymann D, Pratt B, Smolina K, Gladstone EJ, Raymond C (2016). Sex differences in the risk of receiving potentially inappropriate prescriptions among older adults. Age Ageing.

[46] Lopez AD, Collishaw NE, Piha T (1994). A descriptive model of the cigarette epidemic in developed countries. Tob Control.

[47] Huber CA, Szucs TD, Rapold R, Reich O. Identifying patients with chronic conditions using pharmacy data in Switzerland: an updated mapping approach to the classification of medications. BMC Public Health. 2013;13(1). 10.1186/1471-2458-13-1030 PMC3840632 24172142

[48] Ibarra-Castillo C, Guisado-Clavero M, Violan-Fors C, Pons-Vigués M, López-Jiménez T, Roso-Llorach A (2018). Survival in relation to multimorbidity patterns in older adults in primary care in Barcelona, Spain (2010-2014): a longitudinal study based on electronic health records. J Epidemiol Community Health.

