# Supplementary material for: Medication patterns in older adults with multimorbidity: a cluster analysis of primary care patients
Source: BMC Fam Pract. 2019 Jun 13;20:82. doi: 10.1186/s12875-019-0969-9 (PMC6567459; doi:10.1186/s12875-019-0969-9)
Supplement: Supplementary file 1 — The main groups of the Anatomical Therapeutic Chemical (ATC) system. (DOCX 12 kb) [file 12875_2019_969_MOESM1_ESM.docx]

Additional file 1. The main groups of the Anatomical Therapeutic Chemical (ATC) system.

| **Capital letter** | **ATC system** |
| --- | --- |
| A | Alimentary tract and metabolism |
| B | Blood and blood forming organs |
| C | Cardiovascular system |
| D | Dermatologicals |
| G | Genito urinary system and sex hormones |
| H | Systemic hormonal preparations, excl. sex hormones and insulins |
| J | Antiinfectives for systemic use |
| L | Antineoplastic and immunomodulating agents |
| M | Musculo-skeletal system |
| N | Nervous system |
| P | Antiparasitic products, insecticides and repellents |
| R | Respiratory system |
| S | Sensory organs |
| V | Various |
