# Supplementary material for: Medication patterns in older adults with multimorbidity: a cluster analysis of primary care patients
Source: BMC Fam Pract. 2019 Jun 13;20:82. doi: 10.1186/s12875-019-0969-9 (PMC6567459; doi:10.1186/s12875-019-0969-9)
Supplement: Supplementary file 2 — Medication patterns across women 80-94 years attended in primary health centres in Barcelona during 2009 (N = 31,848). Selected criteria: Prevalence ≥20 or Observed/Expected ratio ≥2. (DOCX 24 kb) [file 12875_2019_969_MOESM2_ESM.docx]

Additional file 2. Medication patterns across women 80-94 years attended in primary health centres in Barcelona during 2009 (N= 31,848). Selected criteria: Prevalence ≥20 or Observed/Expected ratio ≥2.

|  |  | **Cluster 1 n=13,538 (43%)** | |  |  |  |
| --- | --- | --- | --- | --- | --- | --- |
|  | **Code^&^** | **Drugs** | | **Pre*** | **O/E ratio^#^** | **Exclus.** |
| **Non-specifc pattern** | A02BC | Proton pump inhibitors | | 27% | 0,53 | 22% |
|  | C10AA | HMG CoA reductase inhibitors | | 26% | 0,74 | 32% |
|  | N02BE | Anilides | | 22% | 0,60 | 26% |
|  | C09AA | ACE inhibitors, plain | | 21% | 0,91 | 39% |
|  | N05BA | Benzodiazepine derivatives | | 20% | 0,67 | 28% |
|  |  |  | |  |  |  |
|  |  | **Cluster 2 n=6,889 (22%)** | |  |  |  |
|  | **Code^&^** | **Drugs** | | **Pre*** | **O/E ratio** | **Exclus.** |
| **"Musculo-skeletal system" and "Alimentary tract and metabolism" and "Nervous system" pattern** | A02BC | Proton pump inhibitors | | 82% | 1.58 | 34% |
|  | N02BE | Anilides | | 64% | 1.78 | 39% |
|  | N05BA | Benzodiazepine derivatives | | 48% | 1.60 | 35% |
|  | C10AA | HMG CoA reductase inhibitors | | 34% | 0.95 | 21% |
|  | B01AC | Platelet aggregation inhibitors excl. Heparin | | 31% | 0.93 | 20% |
|  | M05BA | Bisphosphonates | | 27% | 1.99 | 43% |
|  | N06AB | Selective serotonin reuptake inhibitors | | 26% | 1.59 | 34% |
|  | M02AA | Antiinflammatory preparations, non-steroids for topical use | | 22% | 2.67 | 58% |
|  | C05CA | Bioflavonoids | | 18% | 2.14 | 46% |
|  | M01AE | Propionic acid derivatives | | 16% | 3.12 | 67% |
|  | N02AX | Other opioids | | 13% | 3.05 | 66% |
|  | A02AD | Combinations and complexes of aluminium, calcium and | | 11% | 2.56 | 55% |
|  |  | magnesium compounds | |  |  |  |
|  | M01AX | Other antiinflammatory and antirheumatic agents, | | 9% | 2.70 | 58% |
|  |  | non-steroids | |  |  |  |
|  | N07CA | Antivertigo preparations | | 9% | 2.14 | 46% |
|  | M01AB | Acetic acid derivatives and related substances | | 8% | 3.01 | 65% |
|  | N03AX | Other antiepileptics | | 7% | 2.15 | 46% |
|  | G04BD | Drugs for urinary frequency and incontinence | | 7% | 2.00 | 43% |
|  | A12AA | Calcium | | 7% | 2.34 | 51% |
|  | N02BB | Pyrazolones | | 6% | 3.04 | 66% |
|  | D01AC | Imidazole and triazole derivatives | | 5% | 2.13 | 46% |
|  | A11CC | Vitamin D and analogues | | 5% | 2.36 | 51% |
|  | H02AB | Glucocorticoids | | 5% | 2.48 | 54% |
|  | A03FA | Propulsives | | 5% | 2.41 | 52% |
|  | D07AC | Corticosteroidas, potent (group III) | | 4% | 2.91 | 63% |
|  | N06AA | Non-selective monoamine reuptake inhibitors | | 4% | 2.56 | 55% |
|  | C04AE | Ergot alkaloids | | 3% | 2.05 | 44% |
|  | M05BX | Other drugs affecting bone structure and mineralization | | 3% | 2.11 | 46% |
|  | R06AX | Other antihistamines for systemic use | | 2% | 2.10 | 45% |
|  |  |  | |  |  |  |
|  |  | **Cluster 3 n=5,002 (16%)** | |  |  |  |
|  | **Code^&^** | **Drugs** | | **Pre*** | **O/E ratio** | **Exclus.** |
| **Alimentary tract and metabolism pattern** | B01AC | Platelet aggregation inhibitors excl. Heparin | | 77% | 2.33 | 37% |
|  | C10AA | HMG CoA reductase inhibitors | | 68% | 1.93 | 30% |
|  | A02BC | Proton pump inhibitors | | 67% | 1.29 | 20% |
|  | A10BA | Biguanides | | 37% | 4.15 | 65% |
|  | N02BE | Anilides | | 32% | 0.89 | 14% |
|  | C08CA | Dihydropyridine derivatives | | 31% | 1.85 | 29% |
|  | N05BA | Benzodiazepine derivatives | | 29% | 0.97 | 15% |
|  | C09AA | ACE inhibitors, plain | | 28% | 1.23 | 19% |
|  | C07AB | Beta blocking agents, selective | | 27% | 2.58 | 41% |
|  | C01DA | Organic nitrates | | 26% | 3.75 | 59% |
|  | A10BB | Sulfonylureas | | 25% | 4.23 | 66% |
|  | C03CA | Sulfonamides, plain | | 23% | 1.39 | 22% |
|  | C09CA | Angiotensin II antagonists, plain | | 20% | 1.40 | 22% |
|  | A10AC | Insulins and analogues for injection, intermediate-acting | | 7% | 4.16 | 65% |
|  | A10AE | Insulins and analogues for injection, long-acting | | 7% | 4.73 | 74% |
|  | C02CA | Alpha-adrenoreceptor antagonists | | 6% | 2.45 | 38% |
|  | A10BX | Other blood glucose lowering drugs, excl. Insulins | | 5% | 4.63 | 73% |
|  | C07AG | Alpha and beta blocking agents | | 5% | 2.21 | 35% |
|  |  |  | |  |  |  |
|  |  | **Cluster 4 n=2,740 (9%)** | |  |  |  |
|  | **Code^&^** | **Drugs** | | **Pre*** | **O/E ratio** | **Exclus.** |
| **Cardiovascular system pattern** | B01AA | Vitamin K antagonists | | 72% | 7.75 | 67% |
|  | C03CA | Sulfonamides, plain | | 69% | 4.14 | 36% |
|  | A02BC | Proton pump inhibitors | | 61% | 1.18 | 10% |
|  | C01AA | Digitalis glycosides | | 50% | 8.88 | 76% |
|  | N02BE | Anilides | | 39% | 1.10 | 9% |
|  | C09AA | ACE inhibitors, plain | | 34% | 1.52 | 13% |
|  | C10AA | HMG CoA reductase inhibitors | | 33% | 0.92 | 8% |
|  | N05BA | Benzodiazepine derivatives | | 32% | 1.05 | 9% |
|  | C07AB | Beta blocking agents, selective | | 22% | 2.10 | 18% |
|  | A12BA | Potassium | | 21% | 6.03 | 52% |
|  | C09CA | Angiotensin II antagonists, plain | | 21% | 1.48 | 13% |
|  | C03DA | Aldosterone antagonists | | 15% | 7.05 | 61% |
|  | C01DA | Organic nitrates | | 14% | 2.05 | 18% |
|  | M04AA | Preparations inhibiting uric acid production | | 12% | 2.85 | 25% |
|  | C08DB | Benzothiazepine derivatives | | 11% | 3.09 | 27% |
|  | C07AG | Alpha and beta blocking agents | | 9% | 4.42 | 38% |
|  | C01BD | Antiarrhythmics, class III | | 8% | 4.89 | 42% |
|  | C08DA | Phenyllalkylamine derivatives | | 3% | 2.64 | 23% |
|  |  |  | |  |  |  |
|  |  | **Cluster 5 n=2,007 (6%)** | |  |  |  |
|  | **Code^&^** | **Drugs** | | **Pre*** | **O/E ratio** | **Exclus.** |
| **Nervous system pattern** | A02BC | Proton pump inhibitors | | 56% | 1.09 | 7% |
|  | N06DA | Anticholinesterases | | 48% | 13.70 | 86% |
|  | B01AC | Platelet aggregation inhibitors excl. Heparin | | 46% | 1.39 | 9% |
|  | N06AB | Selective serotonin reuptake inhibitors | | 36% | 2.20 | 14% |
|  | N05BA | Benzodiazepine derivatives | | 35% | 1.17 | 7% |
|  | N02BE | Anilides | | 33% | 0.93 | 6% |
|  | N06DX | Other anti-dementia drugs | | 28% | 7.96 | 50% |
|  | C10AA | HMG CoA reductase inhibitors | | 26% | 0.75 | 5% |
|  | N06AX | Other antidepressants | | 26% | 5.68 | 36% |
|  | N05AH | Diazepines, oxazepines, thiazepines and oxepines | | 22% | 15.16 | 96% |
|  | C09AA | ACE inhibitors, plain | | 22% | 0.95 | 6% |
|  | N05AX | Other antipsychotics | | 21% | 14.38 | 91% |
|  | A06AD | Osmotically acting laxatives | | 17% | 2.38 | 15% |
|  | N04BA | Dopa and dopa derivatives | | 13% | 6.89 | 43% |
|  | N06BX | Other psychostimulants and nootropics | | 6% | 2.76 | 17% |
|  | B03BA | Vitamin B12 (cyanocabalamin and analogues) | | 5% | 2.37 | 15% |
|  |  |  | |  |  |  |
|  |  | **Cluster 6 n=1,672 (5%)** | |  |  |  |
|  | **Code^&^** | **Drugs** | | **Pre*** | **O/E ratio** | **Exclus.** |
| **Respiratory system pattern** | R03AC | Selective beta-2-adrenoreceptor agonists | | 70% | 14.65 | 77% |
|  | R03BB | Anticholinergics | | 63% | 14.13 | 74% |
|  | A02BC | Proton pump inhibitors | | 62% | 1.19 | 6% |
|  | R03AK | Adrenergics in combination with corticosteroids or | | 45% | 9.69 | 51% |
|  |  | other drugs, excl. Anticholinergics | |  |  |  |
|  | N02BE | Anilides | | 44% | 1.22 | 6% |
|  | R03BA | Glucocorticoids | | 43% | 15.41 | 81% |
|  | B01AC | Platelet aggregation inhibitors excl. Heparin | | 37% | 1.13 | 6% |
|  | C10AA | HMG CoA reductase inhibitors | | 33% | 0.92 | 5% |
|  | C03CA | Sulfonamides, plain | | 32% | 1.93 | 10% |
|  | N05BA | Benzodiazepine derivatives | | 31% | 1.03 | 5% |
|  | C09AA | ACE inhibitors, plain | | 26% | 1.16 | 6% |
|  | R05CB | Mucolytics | | 12% | 5.98 | 31% |
|  | A12BA | Potassium | | 8% | 2.13 | 11% |
|  | C08DB | Benzothiazepine derivatives | | 8% | 2.14 | 11% |
|  | C03DA | Aldosterone antagonists | | 5% | 2.32 | 12% |
|  | H02AB | Glucocorticoids | | 4% | 2.18 | 11% |
|  | G04CA | Alpha-adrenoreceptor antagonists | | 4% | 2.44 | 13% |
|  | R06AX | Other antihistamines for systemic use | | 3% | 2.37 | 12% |
|  |  |  | |  |  |  |
|  |  |  | |  |  |  |
| *Code^&:^ chemical subgroup, 4rt level, ATC code (Anatomical Therapeutic Chemical classification) | | | | | | |
|  | from the World Health Organization | |  | |  |  |
| O/E ratio^#^: observed/expected ratio | | |  | |  |  |
| Pre*: Prevalence | |  |  | |  |  |
| Exclus.: Exclusivity | | |  | |  |  |
