# Supplementary material for: Medication patterns in older adults with multimorbidity: a cluster analysis of primary care patients
Source: BMC Fam Pract. 2019 Jun 13;20:82. doi: 10.1186/s12875-019-0969-9 (PMC6567459; doi:10.1186/s12875-019-0969-9)
Supplement: Supplementary file 3 — Medication patterns across men 65-79 years attended in primary health centres in Barcelona during 2009 (N = 41,931). Selected criteria: Prevalence ≥20 or Observed/Expected ratio ≥ 2. (DOCX 26 kb) [file 12875_2019_969_MOESM3_ESM.docx]

Additional file 3. Medication patterns across men 65-79 years attended in primary health centres in Barcelona during 2009 (N= 41,931). Selected criteria: Prevalence ≥20 or Observed/Expected ratio ≥ 2.

|  |  | **Cluster 1 n=20,424 (49%)** |  | |  |  |
| --- | --- | --- | --- | --- | --- | --- |
|  | **Code^&^** | **Drugs** | **Pre*** | | **O/E ratio** | **Exclus.** |
| **Non-specifc pattern** | C10AA | HMG CoA reductase inhibitors | 30% | | 0.70 | 34% |
|  | C09AA | ACE inhibitors, plain | 21% | | 0.89 | 43% |
|  |  |  |  | |  |  |
|  |  | **Cluster 2 n=5,905 (14%)** |  | |  |  |
|  | **Code^&^** | **Drugs** | **Pre*** | | **O/E ratio** | **Exclus.** |
| **Cardiovascular system pattern** | B01AC | Platelet aggregation inhibitors excl. Heparin | 92% | | 2.79 | 39% |
|  | C10AA | HMG CoA reductase inhibitors | 88% | | 2.02 | 28% |
|  | A02BC | Proton pump inhibitors | 65% | | 1.80 | 25% |
|  | C07AB | Beta blocking agents, selective | 51% | | 3.62 | 51% |
|  | C01DA | Organic nitrates | 37% | | 5.21 | 73% |
|  | C09AA | ACE inhibitors, plain | 35% | | 1.49 | 21% |
|  | N05BA | Benzodiazepine derivatives | 23% | | 1.61 | 23% |
|  | C08CA | Dihydropyridine derivatives | 23% | | 1.61 | 23% |
|  | C08DB | Benzothiazepine derivatives | 9% | | 3.01 | 42% |
|  | C10AX | Other lipid modifying agents | 8% | | 4.18 | 59% |
|  | C01EB | Other cardiac preparations | 5% | | 2.36 | 33% |
|  | C07AG | Alpha and beta blocking agents | 5% | | 2.00 | 28% |
|  | A02BA | H2 - receptor antagonists | 4% | | 2.29 | 32% |
|  |  |  |  | |  |  |
|  |  | **Cluster 3 n=5,327 (13%)** |  | |  |  |
|  | **Code^&^** | **Drugs** | **Pre*** | | **O/E ratio** | **Exclus.** |
| **Alimentary tract and metabolism pattern** | A10BA | Biguanides | 65% | | 4.34 | 55% |
|  | C10AA | HMG CoA reductase inhibitors | 58% | | 1.33 | 17% |
|  | B01AC | Platelet aggregation inhibitors excl. Heparin | 55% | | 1.67 | 21% |
|  | A10BB | Sulfonylureas | 46% | | 5.52 | 70% |
|  | A02BC | Proton pump inhibitors | 35% | | 0.96 | 12% |
|  | C08CA | Dihydropyridine derivatives | 26% | | 1.83 | 23% |
|  | C09AA | ACE inhibitors, plain | 24% | | 1.04 | 13% |
|  | S01ED | Beta blocking agents 1 | 17% | | 3.65 | 46% |
|  | S01EE | Prostaglandin analogues 1 | 15% | | 3.46 | 44% |
|  | A10AE | Insulins and analogues for injection, long-acting | 10% | | 5.30 | 67% |
|  | C10AB | Fibrates | 10% | | 2.66 | 34% |
|  | A10BX | Other blood glucose lowering drugs, excl. Insulins | 8% | | 5.46 | 69% |
|  | S01EC | Carbonic anhydrase inhibitors | 6% | | 5.66 | 72% |
|  | A10AC | Insulins and analogues for injection, intermediate-acting | 6% | | 3.07 | 39% |
|  | A10BD | Combinations of oral blood glucose lowering drugs | 5% | | 4.51 | 57% |
|  | A10AD | Insulins and analogues for injection, intermediate- | 4% | | 3.52 | 45% |
|  |  | or long- acting combined with fast- acting |  | |  |  |
|  |  |  |  | |  |  |
|  |  | **Cluster 4 n=5,194 (12%)** |  | |  |  |
|  | **Code^&^** | **Drugs** | | **Pre*** | **O/E ratio** | **Exclus.** |
| **"Musculo-skeletal system" and "Dermatologicals" and "Nervous system" pattern** | A02BC | Proton pump inhibitors | 64% | | 1.76 | 22% |
|  | N02BE | Anilides | 41% | | 3.09 | 38% |
|  | N05BA | Benzodiazepine derivatives | 34% | | 2.36 | 29% |
|  | C10AA | HMG CoA reductase inhibitors | 31% | | 0.70 | 9% |
|  | B01AC | Platelet aggregation inhibitors excl. Heparin | 27% | | 0.81 | 10% |
|  | G04CA | Alpha-adrenoreceptor antagonists | 24% | | 1.40 | 17% |
|  | M01AE | Propionic acid derivatives | 19% | | 5.70 | 71% |
|  | M02AA | Antiinflammatory preparations, non-steroids for topical use | 17% | | 5.26 | 65% |
|  | N06AB | Selective serotonin reuptake inhibitors | 17% | | 2.92 | 36% |
|  | A02AD | Combinations and complexes of aluminium, calcium and | 12% | | 3.73 | 46% |
|  |  | magnesium compounds |  | |  |  |
|  | M01AX | Other antiinflammatory and antirheumatic agents, non- steroids | 11% | | 3.51 | 43% |
|  | M01AB | Acetic acid derivatives and related substances | 11% | | 5.60 | 69% |
|  | N03AX | Other antiepileptics | 10% | | 4.64 | 57% |
|  | A06AC | Bulk-forming laxatives | 9% | | 2.97 | 37% |
|  | D01AC | Imidazole and triazole derivatives | 8% | | 4.64 | 58% |
|  | A06AD | Osmotically acting laxatives | 8% | | 3.42 | 42% |
|  | D07AC | Corticosteroidas, potent (group III) | 8% | | 4.85 | 60% |
|  | N06AX | Other antidepressants | 8% | | 3.85 | 48% |
|  | N02AX | Other opioids | 7% | | 6.53 | 81% |
|  | N05CD | Benzodiazepine derivatives | 7% | | 3.05 | 38% |
|  | G04BD | Drugs for urinary frequency and incontinence | 6% | | 2.48 | 31% |
|  | R05CB | Mucolytics | 6% | | 2.23 | 28% |
|  | C05CA | Bioflavonoids | 5% | | 2.80 | 35% |
|  | M05BA | Bisphosphonates | 5% | | 2.96 | 37% |
|  | N07CA | Antivertigo preparations | 4% | | 3.58 | 44% |
|  | R06AX | Other antihistamines for systemic use | 3% | | 2.82 | 35% |
|  | N05CF | Benzodiazepine related drugs | 3% | | 2.95 | 37% |
|  | C07AA | Beta blocking agents, non-selective | 3% | | 2.52 | 31% |
|  | B03AA | Iron bivalent, oral preparations | 3% | | 2.36 | 29% |
|  | B03BA | Vitamin B12 (cyanocabalamin and analogues) | 2% | | 2.06 | 26% |
|  |  |  |  | |  |  |
|  |  | **Cluster 5 n=3,083 (7%)** |  | |  |  |
|  | **Code^&^** | **Drugs** | **Pre*** | | **O/E ratio** | **Exclus.** |
| **Respiratory system pattern** | R03BB | Anticholinergics | 77% | | 9.78 | 72% |
|  | R03AC | Selective beta-2-adrenoreceptor agonists | 66% | | 10.25 | 75% |
|  | R03AK | Adrenergics in combination with corticosteroids or | 56% | | 8.27 | 61% |
|  |  | other drugs, excl. Anticholinergics |  | |  |  |
|  | A02BC | Proton pump inhibitors | 44% | | 1.21 | 9% |
|  | C10AA | HMG CoA reductase inhibitors | 36% | | 0.83 | 6% |
|  | R03BA | Glucocorticoids | 32% | | 11.1 | 82% |
|  | B01AC | Platelet aggregation inhibitors excl. Heparin | 32% | | 0.95 | 7% |
|  | C09AA | ACE inhibitors, plain | 22% | | 0.94 | 7% |
|  | N02BE | Anilides | 21% | | 1.53 | 11% |
|  | R05CB | Mucolytics | 14% | | 5.50 | 40% |
|  | C08DB | Benzothiazepine derivatives | 7% | | 2.15 | 16% |
|  | H02AB | Glucocorticoids | 4% | | 2.72 | 20% |
|  | R06AX | Other antihistamines for systemic use | 3% | | 2.47 | 18% |
|  |  |  |  | |  |  |
|  |  | **Cluster 6 n=1,998 (5%)** |  | |  |  |
|  | **Code^&^** | **Drugs** | **Pre*** | | **O/E ratio** | **Exclus.** |
| **"Cardiovascular system" and "Alimentary tract and metabolism" pattern** | B01AA | Vitamin K antagonists | 71% | | 9.80 | 47% |
|  | C03CA | Sulfonamides, plain | 57% | | 8.83 | 42% |
|  | C10AA | HMG CoA reductase inhibitors | 54% | | 1.24 | 6% |
|  | A02BC | Proton pump inhibitors | 52% | | 1.43 | 7% |
|  | C01AA | Digitalis glycosides | 38% | | 19.01 | 91% |
|  | C09AA | ACE inhibitors, plain | 36% | | 1.54 | 7% |
|  | B01AC | Platelet aggregation inhibitors excl. Heparin | 24% | | 0.73 | 3% |
|  | C07AB | Beta blocking agents, selective | 24% | | 1.67 | 8% |
|  | C09CA | Angiotensin II antagonists, plain | 22% | | 1.82 | 9% |
|  | M04AA | Preparations inhibiting uric acid production | 22% | | 2.00 | 10% |
|  | C07AG | Alpha and beta blocking agents | 22% | | 9.16 | 44% |
|  | C03DA | Aldosterone antagonists | 21% | | 15.84 | 75% |
|  | C01BD | Antiarrhythmics, class III | 17% | | 13.62 | 65% |
|  | A12BA | Potassium | 14% | | 12.85 | 61% |
|  | C08DB | Benzothiazepine derivatives | 8% | | 2.46 | 12% |
|  | A06AD | Osmotically acting laxatives | 6% | | 2.31 | 11% |
|  | B03AA | Iron bivalent, oral preparations | 6% | | 4.51 | 22% |
|  | A10AC | Insulins and analogues for injection, intermediate-acting | 4% | | 2.33 | 11% |
|  | H02AB | Glucocorticoids | 3% | | 2.00 | 10% |
|  | A10AD | Insulins and analogues for injection, intermediate- | 3% | | 2.40 | 11% |
|  |  | or long- acting combined with fast- acting |  | |  |  |
|  |  |  |  | |  |  |
| *Code^&:^ chemical subgroup, 4rt level, ATC code (Anatomical Therapeutic Chemical classification) | | | | | | |
| from the World Health Organization | | |  | |  |  |
| O/E ratio^#^: observed/expected ratio | | |  | |  |  |
| Pre*: Prevalence | | |  | |  |  |
| Exclus.: Exclusivity | | |  | |  |  |
