# Supplementary material for: Medication patterns in older adults with multimorbidity: a cluster analysis of primary care patients
Source: BMC Fam Pract. 2019 Jun 13;20:82. doi: 10.1186/s12875-019-0969-9 (PMC6567459; doi:10.1186/s12875-019-0969-9)
Supplement: Supplementary file 4 — Medication patterns across men 80-94 years attended in primary health centres in Barcelona during 2009 (N = 12,726). Selected criteria: Prevalence ≥20 or Observed/Expected ratio ≥ 2. (DOCX 23 kb) [file 12875_2019_969_MOESM4_ESM.docx]

Additional file 4. Medication patterns across men 80-94 years attended in primary health centres in Barcelona during 2009 (N= 12,726). Selected criteria: Prevalence ≥20 or Observed/Expected ratio ≥ 2.

|  |  | **Cluster 1 n=4,999 (39%)** |  |  |  |
| --- | --- | --- | --- | --- | --- |
|  | **Code^&^** | **Drugs** | **Pre*** | **O/E ratio** | **Exclus.** |
| **Non-specifc pattern** | B01AC | Platelet aggregation inhibitors excl. Heparin | 22% | 0.50 | 20% |
|  | C09AA | ACE inhibitors, plain | 22% | 0.86 | 34% |
|  | A02BC | Proton pump inhibitors | 21% | 0.46 | 18% |
|  | C10AA | HMG CoA reductase inhibitors | 20% | 0.55 | 22% |
|  |  |  |  |  |  |
|  |  | **Cluster 2 n=2,619 (21%)** |  |  |  |
|  | **Code^&^** | **Drugs** | **Pre*** | **O/E ratio** | **Exclus.** |
| **"Cardiovascular system" and "Alimentary tract and metabolism" pattern** | B01AC | Platelet aggregation inhibitors excl. Heparin | 91% | 2.04 | 42% |
|  | C10AA | HMG CoA reductase inhibitors | 77% | 2.10 | 43% |
|  | A02BC | Proton pump inhibitors | 65% | 1.40 | 29% |
|  | C01DA | Organic nitrates | 36% | 2.85 | 59% |
|  | C09AA | ACE inhibitors, plain | 34% | 1.33 | 27% |
|  | C07AB | Beta blocking agents, selective | 33% | 2.79 | 57% |
|  | C08CA | Dihydropyridine derivatives | 30% | 1.68 | 35% |
|  | A10BA | Biguanides | 28% | 2.46 | 51% |
|  | A10BB | Sulfonylureas | 21% | 2.43 | 50% |
|  | N05BA | Benzodiazepine derivatives | 20% | 1.09 | 22% |
|  | G04CA | Alpha-adrenoreceptor antagonists | 20% | 0.96 | 20% |
|  | A10AE | Insulins and analogues for injection, long-acting | 5% | 2.46 | 51% |
|  | A10AC | Insulins and analogues for injection, intermediate-acting | 5% | 2.36 | 48% |
|  | A10BX | Other blood glucose lowering drugs, excl. Insulins | 4% | 2.30 | 47% |
|  |  |  |  |  |  |
|  |  | **Cluster 3 n=2,150 (17%)** |  |  |  |
|  | **Code^&^** | **Drugs** | **Pre*** | **O/E ratio** | **Exclus.** |
| **"Nervous system" and "Musculo-skeletal system" pattern** | A02BC | Proton pump inhibitors | 71% | 1.53 | 26% |
|  | B01AC | Platelet aggregation inhibitors excl. Heparin | 49% | 1.09 | 18% |
|  | N02BE | Anilides | 43% | 2.05 | 35% |
|  | N05BA | Benzodiazepine derivatives | 35% | 1.95 | 33% |
|  | C10AA | HMG CoA reductase inhibitors | 27% | 0.73 | 12% |
|  | G04CA | Alpha-adrenoreceptor antagonists | 26% | 1.29 | 22% |
|  | N06AB | Selective serotonin reuptake inhibitors | 24% | 2.88 | 49% |
|  | M02AA | Antiinflammatory preparations, non-steroids for topical use | 16% | 2.98 | 50% |
|  | A06AD | Osmotically acting laxatives | 16% | 2.62 | 44% |
|  | M01AE | Propionic acid derivatives | 11% | 4.32 | 73% |
|  | N06DA | Anticholinesterases | 10% | 3.82 | 65% |
|  | A02AD | Combinations and complexes of aluminium, calcium | 10% | 2.42 | 41% |
|  |  | and magnesium compounds |  |  |  |
|  | N06DX | Other anti-dementia drugs | 9% | 3.66 | 62% |
|  | N04BA | Dopa and dopa derivatives | 9% | 3.80 | 64% |
|  | N06AX | Other antidepressants | 9% | 3.77 | 64% |
|  | G04BD | Drugs for urinary frequency and incontinence | 9% | 2.42 | 41% |
|  | N07CA | Antivertigo preparations | 8% | 2.83 | 48% |
|  | N03AX | Other antiepileptics | 8% | 3.06 | 52% |
|  | C05CA | Bioflavonoids | 8% | 2.86 | 48% |
|  | N06BX | Other psychostimulants and nootropics | 8% | 3.61 | 61% |
|  | N05CD | Benzodiazepine derivatives | 7% | 2.40 | 41% |
|  | M01AX | Other antiinflammatory and antirheumatic agents, non-steroids | 7% | 2.79 | 47% |
|  | M01AB | Acetic acid derivatives and related substances | 6% | 3.59 | 61% |
|  | B03BA | Vitamin B12 (cyanocabalamin and analogues) | 6% | 2.25 | 38% |
|  | D07AC | Corticosteroidas, potent (group III) | 5% | 3.00 | 51% |
|  | N02AX | Other opioids | 5% | 3.42 | 58% |
|  | M05BA | Bisphosphonates | 5% | 2.14 | 36% |
|  | D01AC | Imidazole and triazole derivatives | 4% | 2.36 | 40% |
|  | A03FA | Propulsives | 4% | 3.61 | 61% |
|  | H02AB | Glucocorticoids | 4% | 2.02 | 34% |
|  | N05CF | Benzodiazepine related drugs | 3% | 2.02 | 34% |
|  |  |  |  |  |  |
|  |  | **Cluster 4 n=1,329 (10%)** |  |  |  |
|  | **Code^&^** | **Drugs** | **Pre*** | **O/E ratio** | **Exclus.** |
| **Respiratory system pattern** | R03BB | Anticholinergics | 81% | 6.72 | 70% |
|  | R03AC | Selective beta-2-adrenoreceptor agonists | 65% | 7.42 | 77% |
|  | A02BC | Proton pump inhibitors | 54% | 1.17 | 12% |
|  | R03AK | Adrenergics in combination with corticosteroids | 52% | 5.97 | 62% |
|  |  | or other drugs, excl. Anticholinergics |  |  |  |
|  | B01AC | Platelet aggregation inhibitors excl. Heparin | 46% | 1.04 | 11% |
|  | R03BA | Glucocorticoids | 33% | 7.72 | 81% |
|  | C10AA | HMG CoA reductase inhibitors | 32% | 0.87 | 9% |
|  | N02BE | Anilides | 27% | 1.29 | 13% |
|  | C09AA | ACE inhibitors, plain | 25% | 0.98 | 10% |
|  | C03CA | Sulfonamides, plain | 23% | 1.53 | 16% |
|  | G04CA | Alpha-adrenoreceptor antagonists | 23% | 1.12 | 12% |
|  | R05CB | Mucolytics | 18% | 4.67 | 49% |
|  | H02AB | Glucocorticoids | 5% | 2.59 | 27% |
|  |  |  |  |  |  |
|  |  | **Cluster 5 n=1,165 (9%)** |  |  |  |
|  | **Code^&^** | **Drugs** | **Pre*** | **O/E ratio** | **Exclus.** |
| **Cardiovascular system pattern** | B01AA | Vitamin K antagonists | 73% | 5.84 | 53% |
|  | C03CA | Sulfonamides, plain | 65% | 4.28 | 39% |
|  | A02BC | Proton pump inhibitors | 57% | 1.24 | 11% |
|  | C01AA | Digitalis glycosides | 45% | 8.17 | 75% |
|  | C10AA | HMG CoA reductase inhibitors | 41% | 1.11 | 10% |
|  | C09AA | ACE inhibitors, plain | 37% | 1.45 | 13% |
|  | B01AC | Platelet aggregation inhibitors excl. Heparin | 23% | 0.53 | 5% |
|  | C01DA | Organic nitrates | 23% | 1.78 | 16% |
|  | M04AA | Preparations inhibiting uric acid production | 23% | 1.95 | 18% |
|  | N02BE | Anilides | 22% | 1.08 | 10% |
|  | N05BA | Benzodiazepine derivatives | 21% | 1.16 | 11% |
|  | C07AG | Alpha and beta blocking agents | 18% | 5.95 | 54% |
|  | A12BA | Potassium | 16% | 5.88 | 54% |
|  | C03DA | Aldosterone antagonists | 16% | 7.43 | 68% |
|  | C01BD | Antiarrhythmics, class III | 13% | 5.98 | 55% |
|  | H03AA | Thyroid hormones | 5% | 2.34 | 21% |
|  | A11CC | Vitamin D and analogues | 4% | 3.40 | 31% |
|  |  |  |  |  |  |
|  |  | **Cluster 6 n=464 (4%)** |  |  |  |
|  | **Code^&^** | **Drugs** | **Pre*** | **O/E ratio** | **Exclus.** |
| **Sensory pattern** | S01EE | Prostaglandin analogues 1 | 64% | 10.11 | 37% |
|  | S01ED | Beta blocking agents 1 | 51% | 8.97 | 33% |
|  | B01AC | Platelet aggregation inhibitors excl. Heparin | 48% | 1.09 | 4% |
|  | S01EC | Carbonic anhydrase inhibitors | 48% | 26.95 | 98% |
|  | A02BC | Proton pump inhibitors | 45% | 0.98 | 4% |
|  | C10AA | HMG CoA reductase inhibitors | 37% | 1.01 | 4% |
|  | S01EA | Sympathomimetics in glaucoma therapy 1) | 28% | 27.21 | 99% |
|  | N02BE | Anilides | 25% | 1.19 | 4% |
|  | C09AA | ACE inhibitors, plain | 23% | 0.90 | 3% |
|  | C08CA | Dihydropyridine derivatives | 21% | 1.18 | 4% |
|  | A10AC | Insulins and analogues for injection, intermediate-acting | 4% | 2.06 | 8% |
|  |  |  |  |  |  |
|  |  |  |  |  |  |
| *Code^&:^ chemical subgroup, 4rt level, ATC code (Anatomical Therapeutic Chemical classification) | | | | | |
|  | from the World Health Organization | |  |  |  |
| O/E ratio^#^: observed/expected ratio | | |  |  |  |
| Pre*: Prevalence | |  |  |  |  |
| Exclus.: Exclusivity | | |  |  |  |
